# High-Temperature Mechanical and Microstructural Properties of Well Cement Modified with Ethylene-Vinyl Acetate Polymer and Polypropylene Fibers for Geothermal Well Applications

**DOI:** 10.3390/polym17121587

**Published:** 2025-06-06

**Authors:** Shisen Zhao, Kai Qiu, Zhisong Xu

**Affiliations:** 1 State Key Laboratory of Intelligent Construction and Healthy Operation and Maintenance of Deep Underground Engineering, China University of Mining and Technology, No. 1, University Road, Xuzhou 221116, China; zhaoshisen@cumt.edu.cn; 2 School of Mechanics and Civil Engineering, China University of Mining and Technology, Xuzhou 221116, China; xuzs@cumt.edu.cn

**Keywords:** modified well cement, ethylene-vinyl acetate polymer, polypropylene fiber, high-temperature mechanical properties, microscopic pore structure, synergism

## Abstract

The high-temperature performance of well cement is critical for the construction of deep geothermal wells and high-temperature energy storage wells, where mechanical integrity and pore structure stability govern wellbore reliability. To address the strength degradation and structural deterioration of conventional cements under high temperature, the G-class cement was modified by ethylene-vinyl acetate (EVA) polymer and polypropylene fibers (PF), and their impact under various temperatures was explored. Results show that at 600 °C, the compressive strength of modified cement remains above 30 MPa. While the cumulative pore area decreases at 500 °C, a significant increase in larger pores and a major restructuring of the pore network occurs at 600 °C, reflecting the dual effects of high temperature on the pore structure. The modified cement retains structural integrity and excellent mechanical performance up to 400 °C with minimal strength loss, uniform strain distribution, and stable pore structure. At 500 °C and above, it still maintains load-bearing capacity and deformation adaptability, meeting the service requirements for geothermal wells and high-temperature energy storage wells. Even at 600 °C, the reinforcing effect of EVA and PF degradation products slow down crack propagation, ensuring durability in extreme conditions. The research findings lay the foundation for the development of well cement for high-temperature service environments.

## 1. Introduction

With the rapid development of the global economy, the extensive use of traditional energy sources has led to significant environmental challenges [1,2,3]. In response to these issues, the shift toward clean energy sources has gained considerable attention [4,5,6]. Among these, geothermal energy stands out as a promising solution [7,8]. Geothermal energy, harnessed from the Earth’s internal heat, offers several advantages, such as sustainability, low environmental impact, and the potential to provide a reliable and continuous source of energy [9,10]. It is considered an essential part of the clean energy transition, offering a viable option for reducing pollution and mitigating the adverse effects of conventional energy production.

In geothermal energy extraction, geothermal wells are crucial for accessing deep thermal reservoirs and enabling the sustainable production of geothermal energy. As a key component of the wellbore structure, cement slurry plays an essential role in maintaining the integrity and stability of geothermal wells. Specifically, it not only stabilizes the casing but also provides effective isolation between different geological formations, preventing the migration of undesirable fluids and safeguarding the overall stability of geothermal well. With the global demand for energy continuing to rise, the exploration and extraction of geothermal resources have progressively moved into deeper geological layers, a shift that is inevitably exposing cement slurries to more demanding service conditions. In particular, this advancement results in not only higher in situ stresses and increased groundwater penetration pressures but also significantly elevated temperatures, which causes significant degradation in the mechanical properties and pore structure of the cement slurry. Consequently, these factors pose a serious threat to the long-term integrity of the wellbore and inevitably reduce the efficiency of geothermal energy extraction, which highlights the need for improved cement slurry formulations that can withstand such extreme conditions.

Current research on cement slurries primarily focuses on two main areas [11,12]. The first area involves the mechanical behavior and constitutive relations of traditional cement materials under complex environmental conditions [13,14]. Yang et al. [15,16] conducted static and cyclic loading tests on cement slurry at four temperatures and characterized internal microfracture damage with nuclear magnetic resonance (NMR), which guided the evaluation of the fatigue performance of cement slurry. Li et al. [17] conducted CT-based in situ axial compression tests on oil-well cement samples exposed to supercritical CO_2_ at 12 MPa and 70 °C for 30 days and obtained seven sets of typical digital cores at 12 μm/voxel to assess the long-term stability of the CO_2_ address storage program. The second area of research centers on modifying cement slurries to enhance their performance in extreme working environments [18,19,20]. Tang et al. [21] used nano TiO_2_-modified waterborne epoxy resin (NTWER) as an additive to improve the interfacial bonding performance of cement sheaths in oil and gas wells. Fikeni et al. [22] applied silica fume, nanomaterials and inorganic salts to improve the strength of low-density oil-well cement pastes to meet the shallow offshore cementing service environment. Alareqi et al. [23] modified the compressive and shear bond strength of G class oil-well cement by adding metakaolin (MK).

In traditional cement-based materials, both EVA and PF have demonstrated considerable advantages in enhancing service performance [24,25,26]. For example, previous research has shown that EVA can enhance the flexibility and crack resistance of cement [27,28], while PF can improve tensile strength and toughness [29,30]. EVA is known for its ability to enhance ductility, crack resistance, and impermeability in cement by forming a polymer network within the cement matrix, which reduces thermal shrinkage, inhibits the formation of microcracks, and improves the sealing ability of the cement [31,32,33]. On the other hand, PF improve the tensile strength, flexural toughness and fracture resistance of cement, effectively distributing stress and minimizing the formation of cracks under mechanical and thermal stresses [30,34,35]. These modifications offer significant benefits, particularly in enhancing the durability and mechanical properties of cement under extreme conditions, such as those encountered in geothermal wells. In terms of economic feasibility, both EVA and PF are widely available industrial additives with relatively low cost compared to other advanced polymer systems or nanomaterials. Their incorporation into well-cement slurry only accounts for a small fraction of the total material cost, while offering considerable improvements in performance. This cost–performance balance makes them suitable candidates for large-scale geothermal well applications. Other potential additives, such as nanosilica, carbon nanotubes and epoxy resins, were considered during preliminary investigation. However, they were excluded due to higher cost, processing complexity or poor high-temperature compatibility. EVA and PF were ultimately selected due to their proven efficacy, thermal stability, and cost-effectiveness. However, despite the individual advantages of these additives, there is limited research on the combined use of EVA and PF in cement slurries. Furthermore, the mechanisms by which the dual incorporation of EVA and PF influences the high-temperature performance and degradation behavior of cement remain unclear, highlighting the need for further investigation into their synergistic effects under harsh geothermal conditions.

In this study, a novel approach to enhancing the performance of well-cement slurries for deep geothermal wells was proposed by EVA and PF into the cement matrix. The mechanical properties, thermal stability, and durability of the modified cement were evaluated through a series of laboratory tests, providing a comprehensive understanding of its performance under extreme conditions. The effects of EVA and PF incorporation on cement performance were analyzed, with a focus on their synergistic impact on the long-term stability and integrity of the cement in high-temperature geothermal environments. The results of this study contribute to the development of more reliable cement formulations, addressing the challenges posed by the harsh conditions encountered in deep geothermal energy extraction.

## 2. Materials and Methodology

### 2.1. Materials

**Figure 1:**
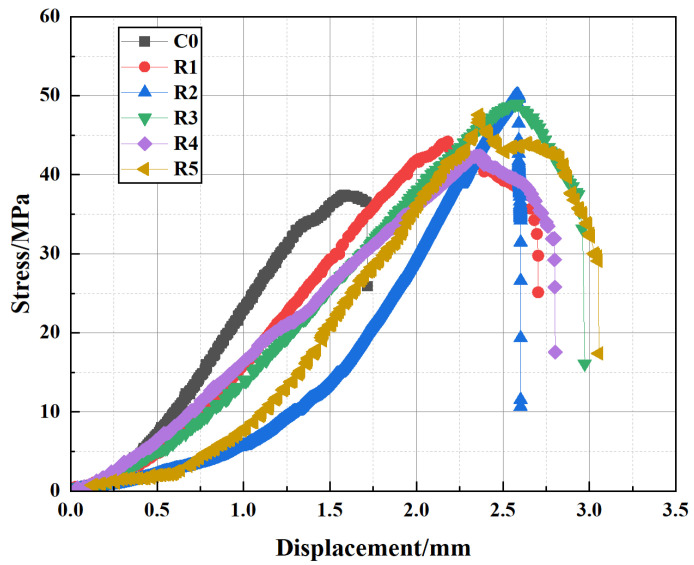
The compressive stress–strain curves of well-cement slurries with different amounts of polymer and additive additions.

In this study, a well-cement slurry was prepared using a mix of Class G oil-well cement and various additives. Class G cement is a basic oil-well cement widely used in petroleum and natural gas exploration and production. The parameters and applicable conditions of Class G cement are based on the API Standard 10A. The X-ray fluorescence (XRF) method was used to determine the chemical composition of the cement with equipment manufactured by Shenzhen Cosmay Company (Shenzhen, China). The chemical composition of the Class G oil-well cement is summarized in Table 1, which includes major components such as CaO (63.10%), SiO_2_ (19.12%), Fe_2_O_3_ (4.78%) and Al_2_O_3_ (4.03%), along with minor components such as MgO, SO_3_, Na_2_O and TiO_2_. The EVA emulsion (DA-102H type) was purchased from Dalian Chemical Company (Dalian, China), with an EVA content of ≥55%. PF was purchased from Tai’an Zhirong Engineering Materials Co., Ltd. (Tai’an, China). Additionally, a water-reducing agent, dispersant and defoamer were included in the formulation to improve the slurry’s fluidity, stability and prevent bubble formation. A series of specimens were prepared with different EVA contents (1–5%) and PF contents (0.1–0.5%). The detailed proportions of these raw materials in the cement slurry formulation are provided in Table 2, and the percentages are based on the mass of the cement. Uniaxial compression tests were performed on these specimens, and the results revealed that the combination of 3% EVA and 0.5% PF (corresponding to approximately 2.7 kg/m^3^) yielded the highest uniaxial compressive strength, as shown in Figure 1. This optimal dosage was then adopted for the subsequent tests. Peng et al. [36] conducted compressive strength tests on polypropylene fiber modified well cement and reported room temperature strengths below 50 MPa. In contrast, the modified cement in our study achieved a compressive strength exceeding 50 MPa at room temperature. This improvement is attributed to the synergistic enhancement provided by the combined use of EVA polymer and PF, which together contributed to improved toughness and matrix integrity.

### 2.2. Sample Preparation

The mixture was prepared using a blade-type mixer with a capacity of 1 L. Initially, Class G oil-well cement was placed in the mixing container, followed by the addition of PF. The mixture was dry mixed for 60 s to achieve uniform distribution of the fibers within the cement matrix. This step is crucial for preventing fiber clumping and ensuring their even dispersion, which is important for maximizing the reinforcement effect of the fibers. Next, water was added to the dry mix, and the mixture was stirred at a low speed (4000 rpm) for 15 s to ensure proper wetting of the cement and the commencement of the hydration process. Subsequently, water-reducing agent and dispersant were added, and the stirring speed was increased to 12,000 rpm for 30 s. This step improves the fluidity of the slurry and ensures uniform distribution of the additives within the mixture. Afterward, the EVA emulsion was gradually added, and the stirring speed was 500 rpm for 60 s to ensure the polymer was well integrated into the slurry, and to prevent the polymer from generating bubbles due to overly fast stirring. Finally, defoamer was added, and the mixture was stirred at 500 rpm for 30 s to remove any bubbles generated during the mixing process, thus reducing porosity and improving the density of the final samples. The prepared slurry was then poured into 50 mm × 100 mm molds for subsequent testing of compressive strength, permeability, and other properties. To minimize the occurrence of air bubbles, the molds were lightly vibrated for 30 s after pouring to ensure uniform filling of the molds. The cured samples were subjected to 28 days of standard curing at room temperature prior to the high-temperature tests. This curing period was chosen to ensure that the cement samples had sufficient time to develop their strength and properties under standard conditions. After this curing period, the samples were then heated to the target temperatures (100 °C, 200 °C, 300 °C, 400 °C, 500 °C, and 600 °C) at a rate of 5 °C/min in a programmable high-temperature oven. The selection of 600 °C as the maximum temperature was based on the findings of previous studies, which have reported temperatures exceeding 500 °C in geothermal wells [37,38,39]. The samples were maintained at the target temperature for 2 h to ensure even heating, after which they were allowed to cool naturally to room temperature in the oven before testing their mechanical properties and microstructural characteristics.

### 2.3. Test Methods

The XRD analysis was conducted using a D8 ADVANCE X-ray diffractometer (Bruker, Germany) equipped with Cu Kα radiation (λ = 1.5405 A), operated at 40 kV and 30 mA. The scanning range was set from 5° to 80° (2θ) with a step size of 0.02° to analyze the crystalline phases of the cementitious matrix. The chemical groups of the modified well-cement slurries were examined by using a Fourier transform infrared (FT-IR) spectrometer (PerkinElmer, Waltham, MA, USA) in the range of 4500–500 cm^−1^. The thermal stability of the cementitious materials was assessed using TGA, which is performed on a TGA/DSC 3+ system (Mettler Toledo, Canton of Zurich, Switzerland). The analysis was conducted under a nitrogen atmosphere with a heating rate of 10 °C/min from room temperature to 800 °C. The weight loss curves were used to determine the dehydration behavior of hydration products and the decomposition of key cementitious phases. The uniaxial compressive strength tests were carried out using the universal mechanical testing machine manufactured by Jinan Shijin Testing Machine Co., Ltd. (Shijin, Jinan, China). The cylindrical cement specimens (*φ*50 mm × 100 mm) were tested at a loading rate of 0.2 mm/min until failure. For each temperature condition, three samples were prepared and tested to ensure the reliability of the results. To capture the strain evolution during compression, a Digital Image Correlation (DIC) system was employed. This system comprised two high-speed cameras (resolution: 2448 × 2048 pixels) and a structured light source. The cameras were positioned perpendicular to each other to ensure full-field strain distribution tracking. Prior to the compression tests, a speckle pattern was applied to the surface of the cement specimens to facilitate accurate displacement and strain calculations. During the loading process, images were recorded at a frequency of 10 Hz. The obtained images were analyzed using Vic-3D software 2024, which allowed for the investigation of strain localization and crack propagation under increasing temperature conditions. The specific parameters for the DIC system, including camera calibration and the speckle pattern preparation, were optimized to ensure the accuracy and reliability of the strain measurements. The microstructure of the cementitious matrix was examined using SEM manufactured by TESCAN Company from the Czech Republic at an accelerating voltage of 10 kV. MIP (Mercury Intrusion Porosimetry) was conducted using an AutoPore IV 9510 mercury porosimeter (TESCAN, Brno, Czech Republic), with a pressure range of 0.1–60,000 psi, allowing the determination of pore sizes from 3 nm to 300 µm. The surface area and pore structure were further analyzed using BET surface area analysis, conducted on a TriStar II 3020 analyzer (Micromeritics, Norcross, GA, USA). These testing methodologies collectively provided a comprehensive evaluation of the thermal, mechanical, and microstructural properties of the modified G-class cement slurry, facilitating a better understanding of its performance under elevated temperature conditions relevant to deep-well applications.

## 3. Result and Discussion

### 3.1. Analysis of XRD, FTIR and Thermal Stability

**Figure 2:**
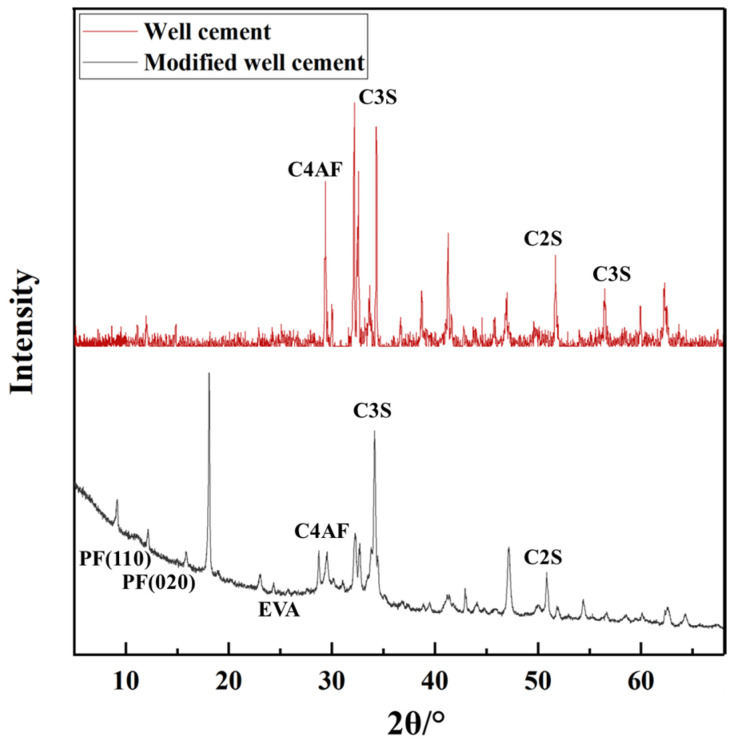
XRD pattern of well cement and modified well cement.

Figure 2 presents the XRD patterns of both the well cement and the modified well-cement slurries. The diffraction peaks clearly indicate the presence of characteristic crystalline phases of G-grade cement, including tricalcium silicate (C3S), dicalcium silicate (C2S), tricalcium aluminate (C3A), and tetracalcium aluminoferrite (C4AF), which are essential for governing the hydration process and mechanical performance of cementitious materials. In the modified well-cement slurry, additional peaks are observed. The presence of EVA is indicated by broad peaks in the low-angle range (approximately 20–30°), attributed to the crystalline segments of EVA arising from the vinyl acetate units. These segments can form ordered structures, leading to detectable diffraction peaks in the XRD pattern. This confirms that EVA is well integrated into the cement matrix. Furthermore, distinct diffraction peaks at 14.2° and 16.3° (2θ) correspond to the (110) and (020) lattice planes of polypropylene fibers (PF), confirming their successful incorporation into the cementitious system. The XRD results demonstrate that the modified cement slurry retains its primary crystalline structure while incorporating EVA and PF. This suggests that the modifiers are effectively integrated without altering the fundamental crystalline phases of the cement, potentially enhancing the mechanical and durability properties of the material. Compared to the unmodified well cement, the modified slurry shows additional peaks associated with EVA and PF, while the main cement phases remain largely unchanged, indicating successful incorporation of the modifiers without disrupting the cement’s inherent crystalline structure.

**Figure 3:**
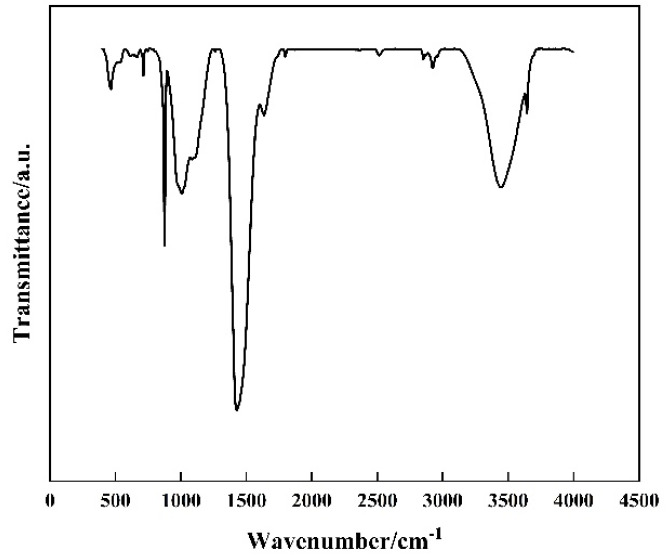
The FTIR spectrum of the modified well cement.

As shown in Figure 3, the FTIR spectrum provided shows several key peaks that can be associated with the components of the modified well cement. The broad peak around 3400 cm^−1^ is characteristic of the O-H stretching vibrations from the hydroxyl groups in the cement hydration products, such as C-S-H gels and calcium hydroxide. The peak around 2900 cm^−1^ corresponds to the C-H stretching vibrations from the methyl and methylene groups in the EVA polymer. The strong peak at approximately 1700 cm^−1^ is attributed to the C=O stretching vibrations from the acetate groups in EVA. Additionally, the peaks around 1600 cm^−1^ and 1400 cm^−1^ can be associated with the bending vibrations of O-H and the deformation of C-H bonds, respectively. The presence of these peaks confirms the incorporation of EVA into the cement matrix. The peak around 1000 cm^−1^ is likely due to the Si-O stretching vibrations from the silicate phases in the cement. The FTIR analysis thus provides evidence of the chemical interactions and the presence of EVA within the cementitious matrix, complementing the XRD results and offering a more comprehensive understanding of the material’s composition and structure.

**Figure 4:**
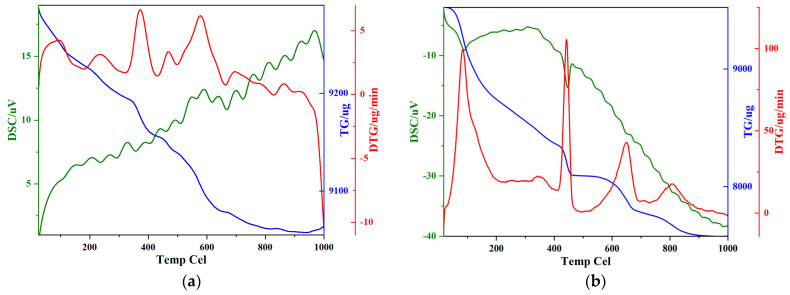
TGA/DTA results of unmodified well-cement slurries and modified well-cement slurries: (**a**) unmodified well-cement slurries; (**b**) modified well cement.

**Figure 5:**
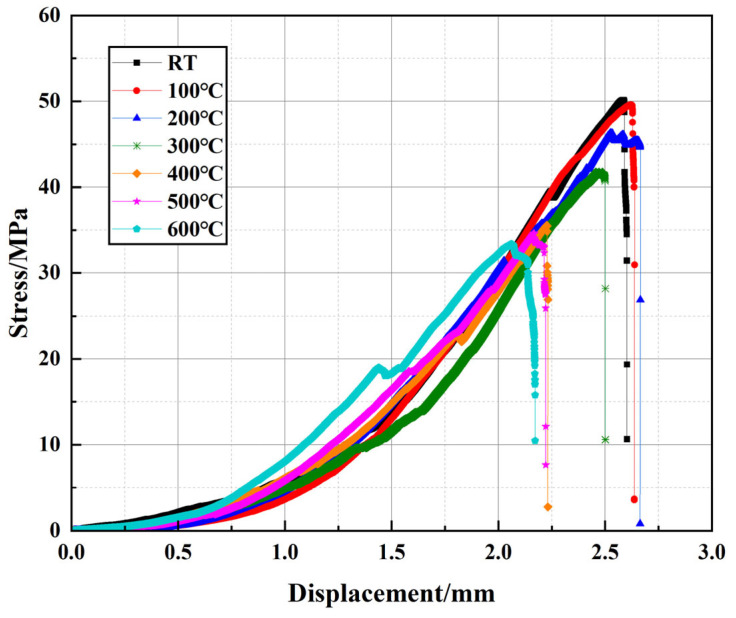
The compressive stress–strain curves of well-cement slurries.

**Figure 6:**
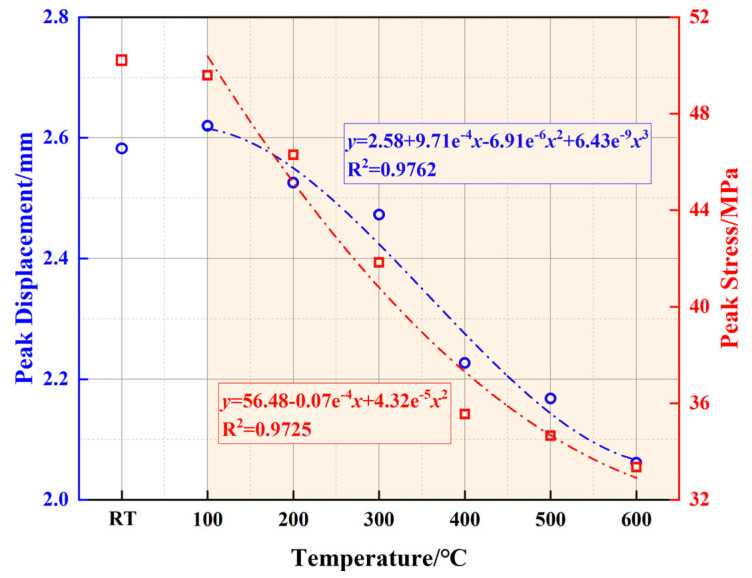
Peak stress and peak displacement of well-cement slurries.

Figure 4 shows the TGA and differential thermal analysis (DTA) results for the cement slurry samples. These analyses provide additional insights into the material’s thermal stability and decomposition behavior at elevated temperatures. The TGA curve indicates a significant mass loss in the temperature range of 100 °C to 200 °C, which can be attributed to the loss of bound water from the hydration products, particularly the C-S-H gel. The DTA curve, which is plotted alongside the TGA data, shows endothermic peaks around 400 °C, which correspond to the thermal decomposition of C-S-H and the release of bound water. At higher temperatures, specifically above 500 °C, the TGA curve shows a sharp decline in mass, which is associated with the decomposition of calcium carbonate (CaCO_3_) and the further degradation of the cement structure. This is also evidenced by the appearance of an additional endothermic peak in the DTA curve, which reflects the loss of carbonates from the cement. The combined TGA and DTA data show that, as the temperature increases, the cement matrix undergoes significant dehydration and decomposition, which leads to an increase in porosity and a loss of structural integrity. This is corroborated by MIP results showing a shift to larger pores (>10 μm) at 600 °C and SEM images depicting severe cracking, while mechanical data (Figure 5 and Figure 6) show a 33.5% strength decline and strain localization.

Taken together, the XRD, FTIR spectrum and TGA/DTA analyses provide a comprehensive understanding of the phase composition and thermal stability of the G-grade cement slurry modified with EVA and PF. The XRD results confirm the presence of essential cementitious phases, along with the successful incorporation of EVA and PF, indicating that these additives are effectively embedded within the cement matrix. The FTIR analysis provides evidence of the chemical interactions and the presence of EVA within the cementitious matrix. Meanwhile, the TGA/DTA results reveal the material’s thermal stability and decomposition behavior, highlighting significant mass loss associated with dehydration of hydration products and polymer degradation. The thermal decomposition of C-S-H and the release of chemically bound water at elevated temperatures suggest that heat exposure could adversely affect the cement’s structural integrity. These findings demonstrate that while the material maintains its phase composition at room temperature, exposure to high temperatures leads to progressive thermal degradation, which must be considered when designing cementing materials for geothermal and deep-well applications.

### 3.2. Analysis of Mechanical Performance

The mechanical performance of well cement under elevated temperatures is a critical factor in ensuring the long-term stability and reliability of cementitious materials in geothermal wells and high-temperature energy storage applications. To investigate the effects of temperature on the compressive behavior of the modified cement slurry, uniaxial compression tests were conducted across a range of temperatures. The stress–strain curves presented in Figure 5 are representative of the typical behavior observed for each temperature condition. At room temperature, the cement exhibits typical linear elastic behavior, characterized by an initial smooth rise in the stress–strain curve as the material deforms elastically. The cement reaches its peak stress, corresponding to the maximum compressive strength, and after this point, the material transitions into the plastic region where strain hardening is observed. At 100 °C, the curve maintains a similar shape but with a slightly reduced slope and peak strength, indicating a minor decrease in elastic modulus and load-bearing capacity. At 300 °C, the decline becomes more pronounced, and the post-peak behavior exhibits a less abrupt failure, suggesting the initiation of thermal degradation within the matrix. At 400 °C, the material shows further reduction in peak strength and an extended plastic region, implying softening of the structure. As the temperature continues to rise, especially at 500 °C and 600 °C, the shape of the stress–strain curve changes more drastically. At these temperatures, the strain increases significantly even after peak stress, and the curves show pronounced ductile behavior, where strain no longer increases linearly but rather exhibits substantial plastic deformation. This change is indicative of severe degradation in the cement’s mechanical properties. The compressive strength of the material decreases markedly, likely due to the high-temperature degradation of hydration products, such as C-S-H gel, which significantly reduces the material’s structural integrity. These temperature-specific changes in both stress–strain response and mechanical strength demonstrate, in a staged and progressive manner, the substantial impact of elevated temperatures on the cement’s performance.

Figure 6 presents the peak stress (*σ_m_*) and corresponding peak displacement (*s_m_*) extracted from Figure 4 to provide a clearer comparison of their trends with temperature variation. Table 3 presents the peak stress and peak displacement values for the modified well cement at various temperatures. And the values reported are the averages of the three samples, ensuring a more accurate representation of the material’s mechanical behavior. Overall, *σ_m_* gradually decreases with increasing temperature, whereas *s_m_* initially increases slightly around 100 °C and then decreases as the temperature rises, indicating a complex evolution of the mechanical behavior of the cementitious matrix under high-temperature conditions. The *σ_m_* reaches 50.15 MPa at RT and decreases to 33.35 MPa at 600 °C, reflecting an overall reduction of approximately 33.5%, demonstrating the influence of elevated temperature on cement strength. In geothermal well design, the cement must be capable of withstanding temperatures up to 200 °C and pressures that can vary significantly depending on the depth and specific application. The compressive strength of the cement is a critical parameter, and it generally needs to be sufficient to maintain the integrity of the wellbore under the expected operational loads and environmental conditions. According to field applications and failure analysis [37,40,41], the minimum compressive strength threshold for wellbore integrity in high-temperature environments (>300 °C) is ≥20 MPa at service temperatures. Therefore, even at 600 °C, the material still maintains a relatively high load-bearing capacity, with a strength exceeding 30 MPa, meeting the mechanical requirements for deep geothermal wells and high-temperature energy storage wells. This result highlights the material’s excellent thermal stability and its capability for long-term service in high-temperature environments. Additionally, compared to traditional cement systems that often experience drastic strength deterioration at 500–600 °C, the modified cement material retains better structural integrity, which can be attributed to the synergistic enhancement effects of EVA and PF that effectively delay the degradation process of the cement matrix. As a result, the material exhibits relatively stable mechanical properties at high temperatures, making it a promising candidate for extreme environments such as deep geothermal and high-temperature energy storage wells. The variation in *s_m_* correlates with the trend observed for *σ_m_*. It increases slightly around 100 °C but subsequently decreases at high temperatures, with an overall reduction of 20.2%. Despite the decline in plastic deformation capacity at high temperatures, the peak displacement remains at 2.06 mm, indicating that the material retains sufficient deformation adaptability to accommodate thermal expansion and stress variations in high-temperature environments.

The changes in mechanical performance primarily result from the phase transitions and decomposition of cement hydration products at high temperatures, with EVA and PF playing crucial roles at different temperature stages. In the temperature range of 100–300 °C, the decline in strength is relatively mild, primarily due to partial dehydration of C-S-H, while its fundamental skeletal structure remains intact. Within this temperature range, EVA forms a flexible phase that enhances the toughness of the cement matrix and improves its crack resistance. Meanwhile, PF exhibit tensile resistance, effectively preventing the propagation of microcracks and enhancing the fracture toughness of the material. However, above 400 °C, the decomposition of Ca(OH)_2_ and the further collapse of the C-S-H structure lead to increased porosity and crack propagation, resulting in a more pronounced strength reduction. As the temperature rises further, EVA gradually decomposes, reducing its interface reinforcement effect and leading to an increase in porosity, which subsequently affects mechanical performance. Nevertheless, even at high temperatures, some degradation products of EVA may still fill microvoids at the microscopic level, mitigating the brittle failure of the cement matrix. Additionally, PF partially carbonize at 400–500 °C, causing a reduction in reinforcement effects, but the remaining fiber skeleton still provides some constraint on crack propagation, thereby slowing down the deterioration rate of the material. Consequently, due to the residual contribution of EVA and the reinforcing effect of PF, the material maintains good load-bearing capacity even at 600 °C, effectively avoiding the severe degradation commonly seen in traditional cement systems at high temperatures and demonstrating strong serviceability under extreme conditions.

To further quantify the variation trends of *σ_m_* and *s_m_* with temperature, curve fitting was performed to establish a mathematical correlation. The relationship between *σ_m_* and temperature follows an exponential function, reflecting the progressive degradation of cement strength due to the thermally induced microstructural changes, decomposition of hydration products, and the deterioration of EVA and PF. Similarly, *s_m_* exhibits a third-order polynomial trend, indicating a complex interplay between temperature, microstructural evolution, and the reinforcing effects of EVA and PF. The R^2^ values for these fitted curves are 0.9725 and 0.9762, respectively, suggesting a strong correlation between temperature and the observed mechanical behavior.

**Figure 7:**
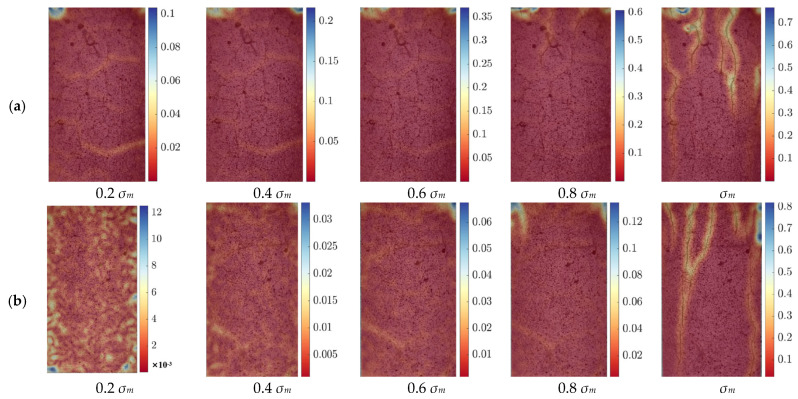

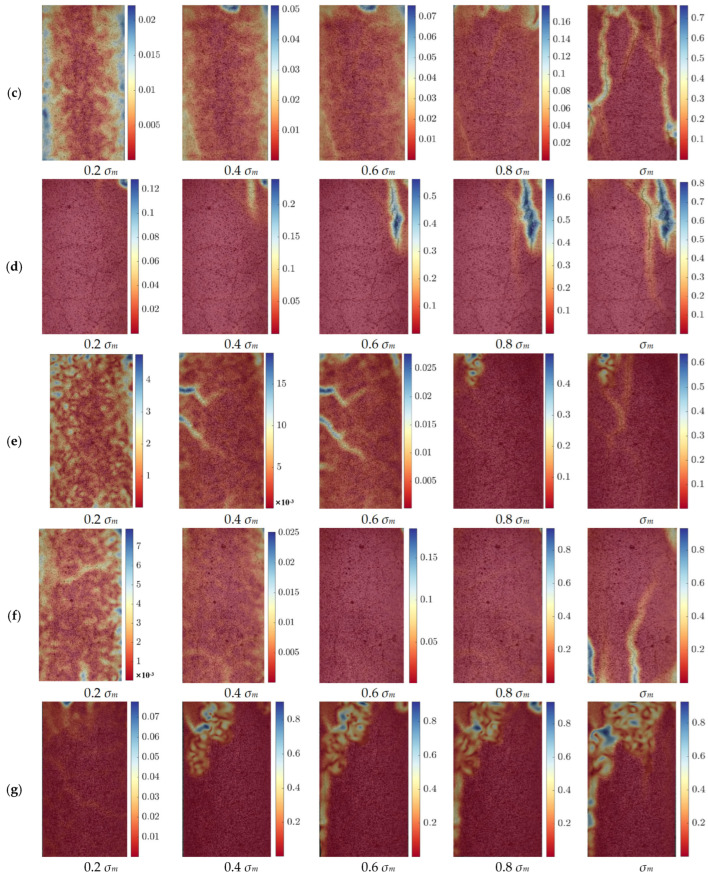
DIC images of well-cement slurries with the strain scale on the right: (**a**) RT; (**b**) 100 °C; (**c**) 2300 °C; (**d**) 300 °C; (**e**) 400 °C; (**f**) 500 °C; (**g**) 600 °C.

Figure 7 illustrates the strain distribution of the cement specimens at different temperatures under progressive loading conditions. The scale on the right side of the figure represents strain, which is a dimensionless quantity. The images correspond to strain measurements at five different stress levels: 0.2 *σ_m_*, 0.4 *σ_m_*, 0.6 *σ_m_*, 0.8 *σ_m_* and *σ_m_*, allowing for a comparative analysis of the strain evolution with increasing external stress. At all temperature levels, the strain distribution exhibits a general pattern: at lower stress levels (0.2 *σ_m_* and 0.4 *σ_m_*), the strain remains relatively uniform across the specimen surface, with only minor localized variations. As the applied stress increases (0.6 *σ_m_* and 0.8 *σ_m_*), strain concentration zones begin to emerge, especially near pre-existing microcracks and weak interfaces within the cement matrix. When the specimens reach the *σ_m_*, clear strain localization occurs, with distinct fracture zones forming, indicating the onset of structural failure. The evolution of these strain fields highlights the progressive damage accumulation in the cement matrix and provides insights into its mechanical response under uniaxial compression.

Despite this common trend across all temperatures, significant differences in strain distribution can be observed between specimens tested at different temperatures. At room temperature and 100 °C, the strain fields remain relatively homogeneous up to 0.8 *σ_m_*, and failure occurs primarily through the formation of a few well-defined crack paths. As the temperature increases to 200 °C and 300 °C, the strain concentration becomes more pronounced, and cracks start to propagate earlier in the loading process, indicating a reduction in the material’s ability to distribute stress evenly. At 400 °C and above, the cement matrix undergoes more severe thermal degradation, and extensive strain localization is evident even at intermediate stress levels (0.6 *σ_m_*). By 500 °C and 600 °C, the material exhibits significantly reduced resistance to deformation, with premature strain concentration and extensive crack formation, leading to an accelerated failure process.

These observed variations in strain distribution can be attributed to the combined effects of cement hydration product decomposition and the thermal responses of EVA and PF. At lower temperatures (<300 °C), the cement matrix retains most of its hydration products, and the EVA enhances toughness by forming a ductile phase within the matrix. Additionally, PF bridge microcracks and help to delay crack propagation, contributing to improved strain distribution. However, as the temperature exceeds 400 °C, EVA starts to degrade, producing small molecular weight hydrocarbons such as ethylene and vinyl acetate, reducing its toughening effect. Meanwhile, the PF begin to carbonize, with their molecular chains breaking down and their strength and flexibility being compromised. The decomposition of C-S-H gel and the breakdown of Ca(OH)_2_ further lead to pore expansion and microstructural deterioration, reducing the material’s ability to accommodate strain effectively. Consequently, at 500 °C and 600 °C, the cement matrix becomes more brittle, strain localizations intensify, and the failure process accelerates. The DIC analysis confirms that the strain distribution and failure behavior of the modified cement material are strongly influenced by temperature. While the incorporation of EVA and PF helps improve the cement’s mechanical performance at lower temperatures, their effectiveness diminishes as thermal degradation progresses.

**Figure 8:**
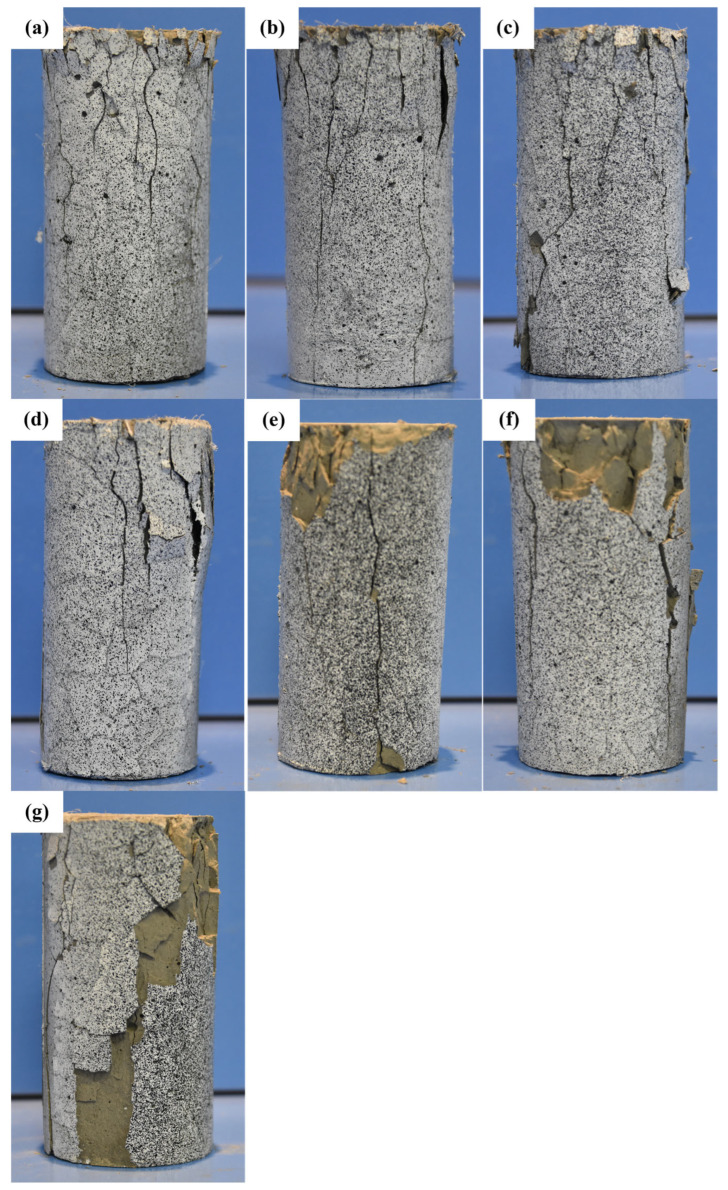
Post-peak failure morphology of cement specimens under different temperatures: (**a**) RT; (**b**) 100 °C; (**c**) 200 °C; (**d**) 300 °C; (**e**) 400 °C; (**f**) 500 °C; (**g**) 600 °C.

Figure 8 presents the final failure morphology of the specimens after uniaxial compression testing at different temperature conditions. As the temperature increases, the failure mode of the specimens evolves, characterized by changes in crack distribution, spalling severity, and overall structural integrity. In the temperature range from room temperature (RT) to 300 °C, the primary failure mode is surface cracking. The cracks are relatively fine and evenly distributed, with no significant spalling observed, indicating that the material retains good integrity and strong load-bearing capacity. During loading, cracks primarily propagate along the axial direction, and their density increases slightly with temperature but does not lead to severe structural failure. This suggests that the material exhibits good thermal stability within this temperature range. At 400 °C, the failure morphology undergoes a noticeable transformation. The number of cracks increases significantly, and localized spalling begins to appear, especially at the top and side surfaces of the specimens. This change indicates that, within this temperature range, the pore structure of the cementitious matrix has undergone significant evolution. The decomposition of Ca(OH)_2_ leads to increased internal porosity, while the partial collapse of the C-S-H structure reduces the overall stiffness of the material, accelerating crack propagation. As the temperature further rises to 500 °C and above, spalling becomes more severe, and cracks begin to penetrate deeper into the specimen, causing extensive delamination, particularly in the highly stressed top and side regions. This phenomenon is closely related to the degradation of EVA. Between 400 °C and 500 °C, EVA gradually decomposes, reducing the toughness of the matrix and making it more susceptible to crack propagation and surface fragmentation. Additionally, the thermal degradation of PF at high temperatures further affects the material’s crack resistance. While PF effectively suppress crack propagation at lower temperatures, their reinforcing effect diminishes above 500 °C, accelerating the expansion of cracks. At 600 °C, under extreme thermal conditions, the specimens exhibit through-cracks and significant spalling, but they still maintain a degree of structural integrity without complete disintegration or powdering. This indicates that the modified cementitious material retains a certain level of load-bearing capacity even at high temperatures. This can be attributed to the synergistic effects of EVA and PF. Although EVA undergoes decomposition, its residual degradation products may still fill some micropores at the microstructural level, mitigating the brittle failure of the cement matrix. Additionally, the carbonized remnants of PF provide a skeletal support effect, limiting further crack propagation. As a result, the material maintains relatively stable mechanical performance even at 600 °C.

### 3.3. Analysis of Pore Structure and Permeability

**Figure 9:**
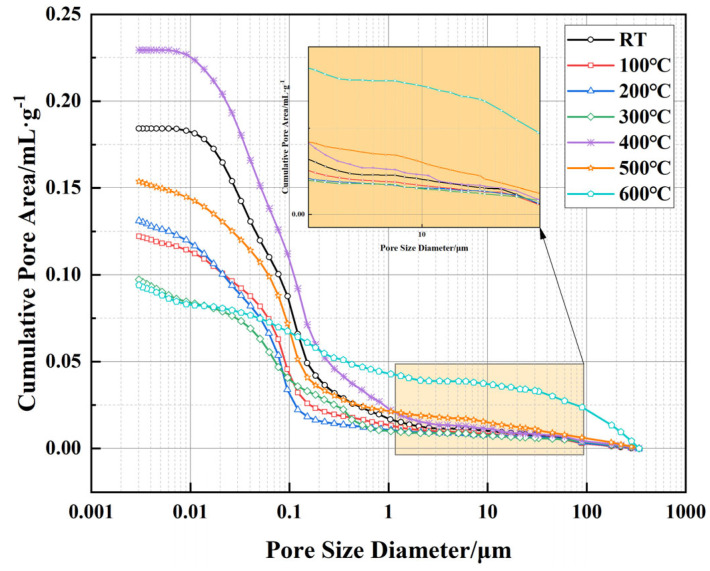
Evolution of cumulative pore area and pore size distribution of well-cement slurries under different temperatures.

Figure 9 presents the relationship between cumulative pore area and pore size of well-cement slurries under different temperature conditions, as measured by MIP technology. This testing method utilizes the ability of mercury to penetrate material pores under varying pressures, allowing for the measurement of pore size distribution and cumulative pore area to reveal the effect of temperature on the pore structure. The results indicate that as temperature increases, the pore structure of the material undergoes significant changes. At room temperature, the cumulative pore area remains relatively low, with pores in the sub-micron to low-micron range dominating the pore distribution. As the temperature rises to 100 °C and 200 °C, the cumulative pore area remains largely stable, suggesting minimal changes in the overall pore structure of the material. However, at 300 °C, a slight decrease in cumulative pore area is observed, which may be attributed to the thermal compaction effect and the collapse of some micropores within the cement matrix. At higher temperatures (400–600 °C), the changes in cumulative pore area become more pronounced, with the most significant reduction occurring at 600 °C. This decline suggests substantial alterations in the pore structure, likely due to the decomposition of cement hydration products (such as C-S-H and Ca(OH)_2_), leading to localized densification. However, this densification process is accompanied by the formation of larger pores. As shown in the inset of Figure 8, a noticeable shift in pore size distribution is observed, with the pore structure at 600 °C showing a clear shift toward larger pores, extending into the high-micron range (10–100 μm and beyond). This indicates that, despite the overall reduction in cumulative pore area, the internal pore network has undergone significant reconstruction. The variations in cumulative pore area and pore size distribution reflect the dual effects of high temperature on the pore structure of the material. In the lower temperature range (RT-300 °C), the pore structure remains relatively stable, with some micropores being reduced due to thermal compaction effects. However, above 400 °C, the decomposition of cement phases and the degradation of the EVA matrix lead to more significant changes in pore structure, gradually shifting toward larger pores. This phenomenon could affect the permeability of the material, particularly between 500 °C and 600 °C, where the increase in large pores suggests changes in pore connectivity, potentially influencing the long-term sealing performance of the material in geothermal wells and high-temperature energy storage applications.

**Figure 10:**
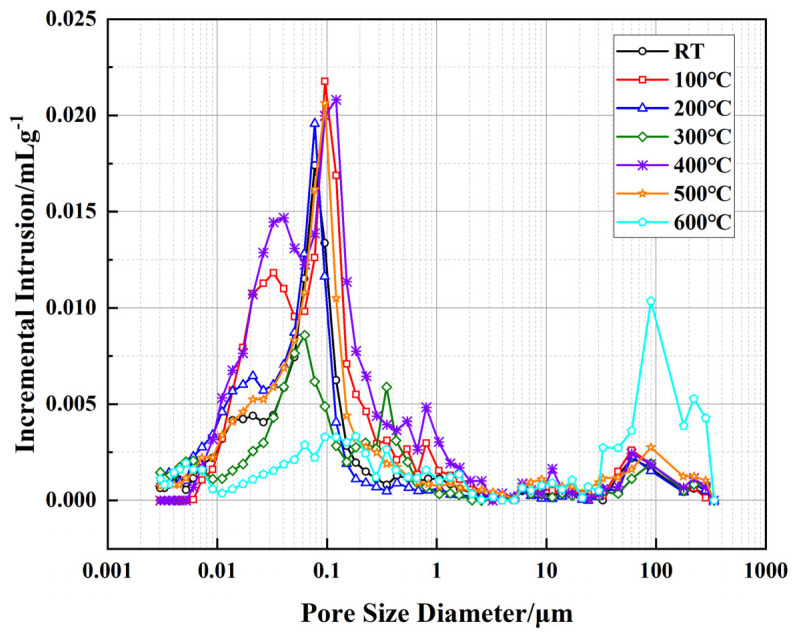
Evolution of incremental intrusion and pore size distribution of well-cement slurries under different temperatures.

Figure 10 illustrates the relationship between incremental intrusion volume and pore size of the well-cement slurries at different temperatures, as measured by MIP technology. This test reflects the volumetric distribution of pores within different size ranges, providing insights into the pore structure evolution of the cementitious matrix. Compared to the cumulative pore area shown in Figure 8, this figure further reveals the trends in pore size distribution, particularly the proportion of micropores and macropores at varying temperature conditions. Overall, from room temperature to 300 °C, the variation in incremental intrusion volume is relatively small, especially in the pore size range between 0.01 and 1 μm, indicating that the micropore structure of the cement matrix remains relatively stable within this temperature range, with no significant changes in pore size distribution. However, at temperatures above 400 °C, a substantial increase in incremental intrusion volume is observed in the larger pore size range, particularly between 80 and 150 μm, with the most pronounced increase occurring at 600 °C. This suggests significant pore structure reconstruction within the cement matrix at elevated temperatures. When analyzed in conjunction with the cumulative pore area data in Figure 8, it is evident that although the overall cumulative pore area decreases at high temperatures, the proportion of larger pores increases while that of micropores decreases. This phenomenon is closely related to the decomposition of cement hydration products, particularly the decomposition of Ca(OH)_2_ between 400 °C and 500 °C and the further collapse of the C-S-H structure, which converts some isolated pores into interconnected ones, thereby affecting the permeability of the material.

Further analysis reveals that the effects of EVA and PF on pore structure vary across different temperature stages. In the range of 100–300 °C, the flexible phase of EVA remains intact, contributing to the maintenance of matrix compactness, while PF play a bridging role in suppressing crack propagation, preventing drastic changes in the pore structure. However, at temperatures above 400 °C, EVA begins to degrade, leading to an increase in internal porosity, while PF gradually undergo carbonization above 500 °C, reducing their ability to constrain pore structure. These factors collectively contribute to the increase in the proportion of larger pores at high temperatures, causing an overall shift in pore distribution towards macropores and consequently affecting the permeability of the material.

**Figure 11:**
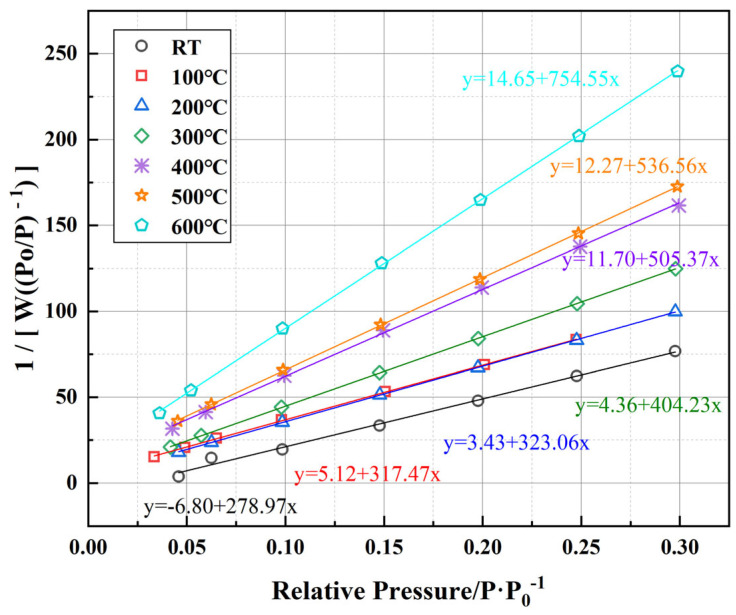
Multi-point BET linear fitting plot of well-cement slurry at different temperatures.

**Figure 12:**
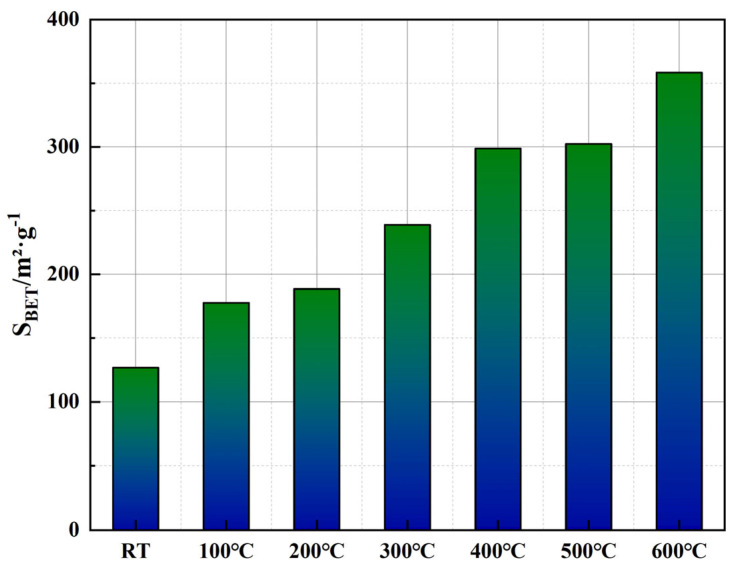
BET-specific surface area evolution of well-cement slurry at different temperatures.

Figure 11 shows the linear fitting curves, based on the BET theory, used to measure the specific surface area of cement samples at different temperatures. The BET method is a classic physical adsorption model that calculates the specific surface area by measuring the amount of nitrogen adsorbed onto the surface of solid materials. The horizontal axis in Figure 11 represents the relative pressure (*P*/*P*_0_), and the vertical axis is the transformed data of the BET linear equation (1/[*W*(*P*/*P*_0_) − 1]). All data sets demonstrate excellent linearity with fitting coefficients exceeding 0.98, indicating that the BET model effectively describes the adsorption behavior of the cement matrix under various temperature conditions. Figure 12 illustrates the BET specific surface area (*S_BET_*) calculated using parameters derived from the linear fitting curves in Figure 11. The calculation involves determining the monolayer adsorption capacity (*W_m_*), which can be obtained from the slope and intercept of the linear regression and computed using Equations (1) and (2).

(1) $$S_{BET}=\frac{W_{m}N_{A}L}{M},$$

where *N_A_* is Avogadro’s number; *L* is the area occupied by a single nitrogen molecule; *M* is the molar mass of nitrogen; *W_m_* is the monolayer adsorption capacity, which is calculated using Equation (2).

(2) $$W_{m}=\frac{1}{k+a},$$

where *k* and *a* are the corresponding parameters of the fitted curves in Figure 11.

The data presented in Figure 12 show a clear and substantial increase in the BET surface area with rising temperature. Initially, the specific surface area at room temperature is 126.83 m^2^/g, indicating the predominance of smaller, microporous structures within the cement matrix. As the temperature increases to 300 °C, *S_BET_* significantly rises by approximately 88.3%, reaching 238.76 m^2^/g, likely due to the generation and propagation of microcracks within the cementitious matrix, resulting from partial dehydration of hydration products such as the C-S-H gel. With further temperature elevation above 400 °C, the increase in surface area becomes even more pronounced, ultimately reaching 358.44 m^2^/g at 600 °C, representing an approximate increase of 182.7% compared to room temperature. This marked elevation at higher temperatures can be attributed to the thermal decomposition of calcium hydroxide (Ca(OH)_2_) and the progressive degradation of the EVA phase, resulting in enhanced exposure of internal pore structures and the formation of larger interconnected pores. Additionally, partial carbonization and structural changes in PF at temperatures above 400 °C likely contribute to maintaining structural integrity of pore networks, thereby sustaining the observed rise in surface area.

Correlating these findings with the pore structure evolution depicted in Figure 9 and Figure 10, it becomes evident that while cumulative pore area exhibits an overall reduction at elevated temperatures, the BET surface area continues to increase, indicating a structural transformation from predominantly closed micropores to more open, interconnected meso- and macroporous systems. Such transitions could significantly impact the permeability characteristics of the cementitious material, particularly at temperatures above 500 °C, where enhanced connectivity between larger pores may alter the material’s long-term sealing performance.

**Figure 13:**
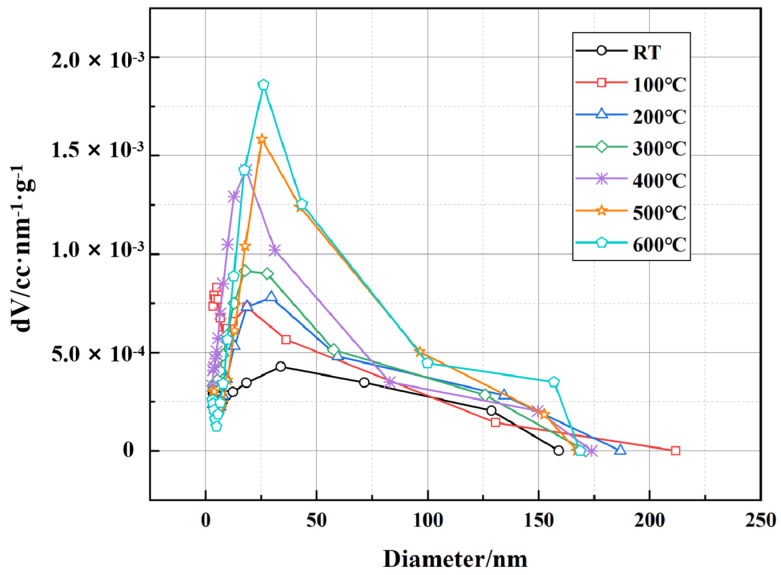
Pore size distribution of well-cement slurry at different temperatures based on BJH method.

Figure 13 presents the pore volume distribution as a function of pore diameter, obtained using the Barrett-Joyner-Halenda (BJH) method. This analysis primarily characterizes the mesopore and macropore structures in the cement matrix. The results indicate that the pore volume distribution varies significantly with increasing temperature. At room temperature, the total pore volume is relatively low, with a dominant peak in the range of 10–50 nm, suggesting a well-packed microstructure. As the temperature rises to 100 °C and 200 °C, a slight increase in pore volume is observed, likely due to the evaporation of physically bound water, leading to partial pore expansion. At higher temperatures (300–400 °C), the peak shifts towards larger pore diameters, and the overall pore volume further increases, indicating the progressive degradation of cement hydration products, particularly the dehydration of calcium silicate hydrate (C-S-H) and the decomposition of portlandite (Ca(OH)_2_). At 500 °C and 600 °C, the pore structure undergoes a more drastic transformation, with a notable increase in larger pores (>100 nm). This suggests that the material experiences substantial microstructural deterioration, accompanied by crack formation and coalescence, which may affect its long-term mechanical performance and permeability in high-temperature applications.

**Figure 14:**
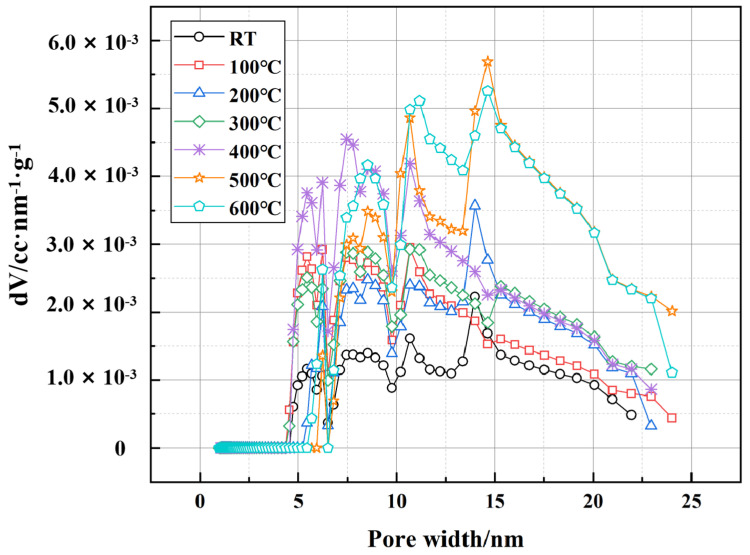
Pore size distribution of well-cement slurry at different temperatures based on DFT method.

Figure 14 illustrates the pore volume distribution derived from the Density Functional Theory (DFT) method, focusing on micropores and small mesopores (pore width < 30 nm). The DFT analysis provides a more detailed assessment of the fine pore structure evolution under thermal exposure. At RT, the dominant pore width is centered around 10 nm, with a relatively narrow distribution. As the temperature increases to 100 °C and 200 °C, a slight increase in pore volume is detected within the 10–20 nm range, indicating that thermal expansion leads to minor structural changes. At 300 °C and 400 °C, a more pronounced shift is observed, with an increased presence of pores in the 15–25 nm range. This can be attributed to the progressive dehydration and partial breakdown of hydration products. The most significant change occurs at 500 °C and 600 °C, where the peak of pore width distribution broadens significantly, and the volume of mesopores increases. The formation of larger mesopores at these temperatures suggests severe matrix deterioration, which is closely related to the thermal degradation of EVA and PF. The decomposition of EVA results in the loss of polymer-filling effects, while the carbonization of PF reduces their ability to bridge microcracks, further accelerating pore coalescence and structural weakening. Nevertheless, microscopic observations reveal that partial EVA residues and PF fragments remain embedded in the matrix even at 600 °C, continuing to contribute to crack mitigation and pore stabilization. These residual effects play a crucial role in maintaining a certain level of structural integrity and mechanical performance under extreme thermal conditions, demonstrating the modified cement’s durability and suitability for high-temperature well applications.

Comparing Figure 13 and Figure 14, it is evident that temperature influences both the mesopore and micropore structures. The BJH results show an overall increase in pore volume with rising temperature, particularly in the mesopore-macropore transition region (>50 nm), whereas the DFT analysis reveals a shift in the fine pore structure towards larger mesopores. These findings indicate that thermal exposure leads to significant changes in the pore network, affecting the permeability and durability of the cementitious matrix.

### 3.4. Microstructural Evolution and Mechanism Analysis Based on SEM

**Figure 15:**
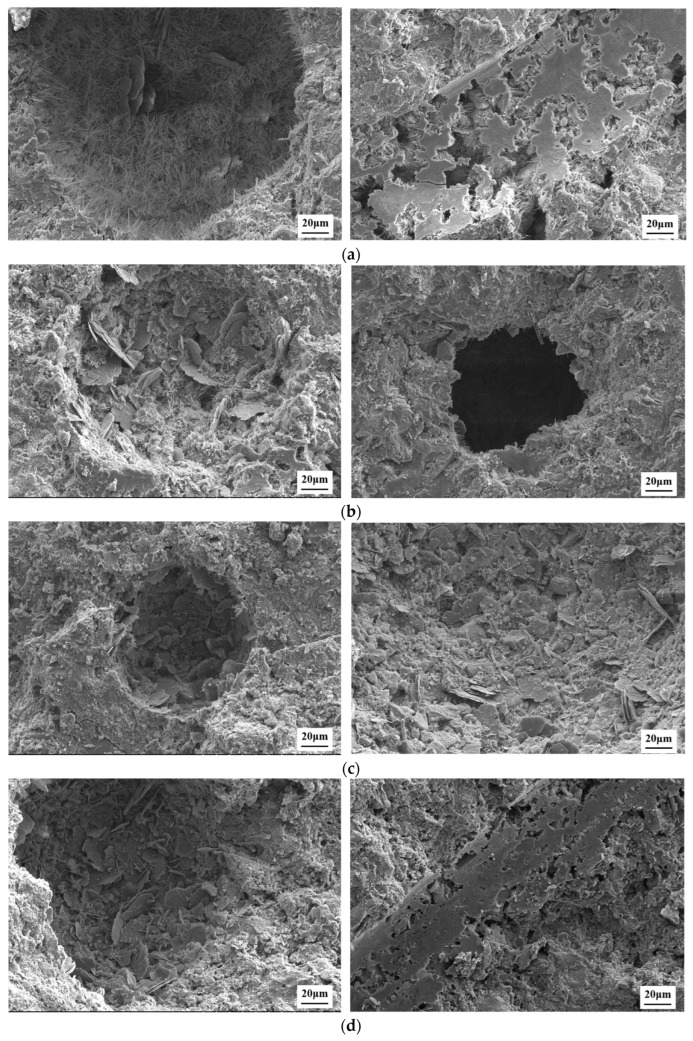

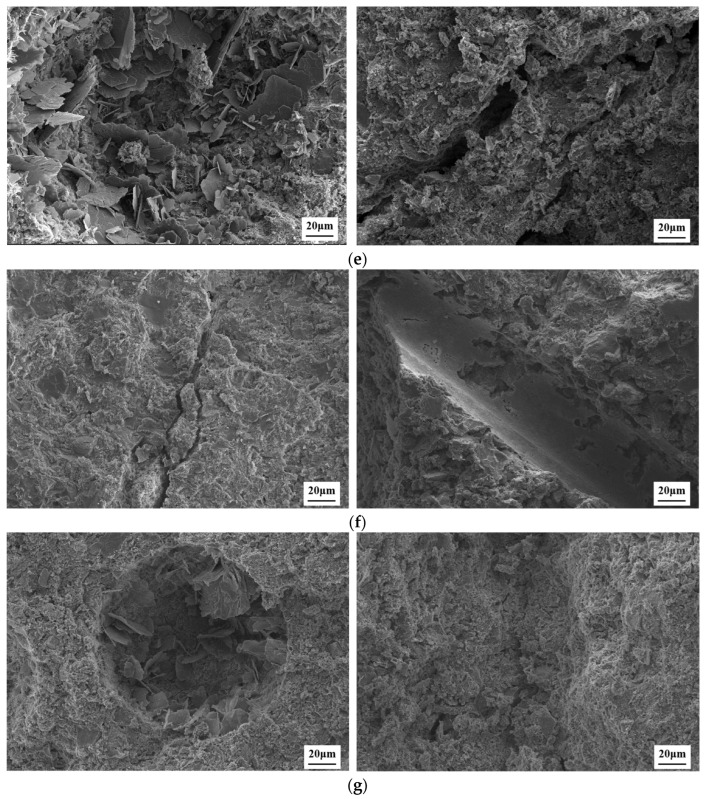
SEM images of well cement at different temperatures: (**a**) RT; (**b**) 100 °C; (**c**) 200 °C; (**d**) 300 °C; (**e**) 400 °C; (**f**) 500 °C; (**g**) 600 °C.

Figure 15 presents the SEM images of the cementitious matrix at different temperatures, providing insights into the microstructural evolution of the material. These images reveal the morphological changes in hydration products, the degradation behavior of EVA, and the structural integrity of PF at elevated temperatures, which collectively influence the mechanical properties and pore structure. At room temperature, the microstructure exhibits a dense and compact cementitious matrix with well-developed needle-like and plate-like C-S-H gel. The hydration products are uniformly distributed, and the interfaces between EVA, PF, and the cement matrix appear well-integrated, contributing to the overall mechanical integrity. The presence of fine and uniformly dispersed pores suggests a low porosity, which is consistent with the high compressive strength observed at RT. Additionally, PF remain intact, effectively bridging cracks and providing reinforcement to the matrix.

As the temperature increases to 100 °C and 200 °C, minor microstructural changes begin to emerge. The C-S-H gel undergoes partial dehydration, leading to a slight increase in porosity. The EVA maintains its integrity at these temperatures but starts softening, slightly affecting the interfacial bonding between the polymer and the cement matrix. The PF remain embedded within the matrix, continuing to restrain microcrack propagation. However, some microvoids appear at the fiber-matrix interfaces, indicating the initial stages of polymer degradation. At 300 °C, the microstructure exhibits more pronounced changes. The dehydration of C-S-H becomes more evident, leading to an increase in porosity and microcrack formation. The EVA begins to degrade, weakening its crack-bridging capability, and interfacial debonding between the polymer and the cement matrix becomes apparent. The PF still provide reinforcement, but their surfaces appear rougher, suggesting the onset of thermal degradation. This microstructural evolution correlates with the observed reduction in mechanical strength and increased pore connectivity.

At 400 °C, significant degradation occurs in both the cement matrix and the reinforcing components. The decomposition of Ca(OH)_2_ leads to increased internal porosity and microcracking, compromising the structural integrity. The EVA undergoes severe decomposition, resulting in a loss of its toughening effect. The PF begin to char and degrade, reducing their reinforcement efficiency. These changes contribute to the notable decline in compressive strength and the formation of larger, more interconnected pores. At 500 °C and 600 °C, the microstructural deterioration intensifies. The cementitious matrix experiences severe crack propagation and pore expansion due to the collapse of the C-S-H gel network. The remaining EVA is almost completely degraded, leaving behind voids that further increase porosity. The PF are mostly carbonized, and their crack-bridging function is largely lost. At 600 °C, the material exhibits the most significant microstructural damage, with large, irregular pores and extensive cracks throughout the matrix. However, despite this severe degradation, the cementitious matrix still retains some degree of integrity, suggesting that the modified material maintains a certain level of mechanical performance under extreme conditions.

Combining mechanical performance, pore structure analysis, and microscopic observations, the EVA and PF fiber-modified well cement can maintain excellent structural integrity and mechanical properties at temperatures up to 400 °C, exhibiting minimal strength loss, uniform strain distribution, and a relatively stable pore structure. At 500 °C and beyond, although the cement matrix undergoes strength reduction and pore structure evolution, the material retains a certain load-bearing capacity and deformation adaptability without experiencing severe brittle failure, thus meeting the service requirements for geothermal wells and high-temperature energy storage wells. Even at 600 °C, the material maintains a compressive strength of over 30 MPa, and the residual reinforcement effect of PF, along with the pore-filling contribution of EVA degradation products, helps mitigate crack propagation and pore deterioration, ensuring a certain degree of durability under extreme high-temperature conditions. Therefore, this material can be regarded as a potential candidate for well cement applications at temperatures up to 500 °C, with further optimization possible to enhance its thermal stability and mechanical performance at even higher temperatures.

## 4. Conclusions

This study investigates the effects of ethylene-vinyl acetate polymer (EVA) and polypropylene fibers (PF) on the performance of G-class well cement under various high-temperature conditions. The mechanical properties, pore structure and microstructural evolution of the modified cement were systematically analyzed. The principal findings of the study are as follows:

(1) The mechanical performance of the modified cement shows a clear temperature dependence. As the temperature increases, the compressive strength decreases, with a 33.5% reduction from 50.15 MPa at room temperature to 33.35 MPa at 600 °C. However, despite the reduction in strength, the material still maintains the relatively excellent load-bearing capacity.

(2) Temperature significantly affects the pore structure of the cement. At 500 °C, the cumulative pore area decreases slightly, but at 600 °C, the proportion of larger pores increases significantly, and the pore network undergoes noticeable restructuring. This is mainly due to the decomposition of cement hydration products and the degradation of the EVA.

(3) SEM analysis reveals significant microstructural changes with increasing temperature. At room temperature, the cement matrix is dense and well-structured, with C-S-H gel forming a robust network. As temperature rises, C-S-H gel undergoes dehydration and degradation, and EVA gradually decomposes, leading to interfacial debonding. Despite this, PF continue to provide reinforcement, mitigating crack propagation.

(4) Combining mechanical performance, pore structure, and microstructural observations, the modified cement retains good stability at extreme temperatures. Below 400 °C, the material exhibits excellent mechanical properties and a stable pore structure. At 500 °C and above, although there is a reduction in strength and restructuring of the pore network, the modified cement still maintains sufficient load-bearing capacity and deformation adaptability, fulfilling the service requirements for high-temperature applications.

## Figures and Tables

**Table 1:** Composition of class G oil-well cement.

Chemical Composition	Relative Content (%)	Chemical Composition	Relative Content (%)
CaO	63.10	TiO_2_	0.277
SiO_2_	19.12	P_2_O_5_	0.0547
Fe_2_O_3_	4.78	MnO	0.0428
Al_2_O_3_	4.03	SrO	0.0477
MgO	3.68	ZnO	0.199
SO_3_	3.64	BaO	/
K_2_O	0.654	ZrO_2_	0.0166
Na_2_O	0.223	Cl	0.0265

**Table 2:** Mixing proportions of well-cement slurries.

Specimen Number	Water-Cement Ratio	EVA	Dispersant	Defoamer	Water Reducer	PF
C0	0.35	/	/	/	0.5%	/
R1	0.35	1%	0.1%	0.3%	0.5%	0.5%
R2	0.35	3%	0.1%	1%	0.5%	0.5%
R3	0.35	5%	0.1%	1.6%	0.5%	0.5%
R4	0.35	3%	0.1%	1%	0.5%	0.1%
R5	0.35	3%	0.1%	1%	0.5%	0.3%

**Table 3:** Peak stress and peak displacement of modified well-cement slurries at various temperatures.

Temperature (°C)	Peak Stress (MPa)	Peak Displacement (mm)
25	50.15	2.59
100	49.58	2.62
200	46.29	2.53
300	41.83	2.47
400	35.55	2.23
500	34.66	2.17
600	33.35	2.06

## Data Availability

Data will be made available on request.
